# Human Ghrelin Improves Vascular Integrity and Survival After Total Body Irradiation

**DOI:** 10.3390/cells15070586

**Published:** 2026-03-26

**Authors:** Wayne Chaung, Asha Jacob, Zhimin Wang, Weng Lang Yang, Max Brenner, Ping Wang

**Affiliations:** 1 Center for Immunology and Inflammation, Feinstein Institutes for Medical Research, Manhasset, NY 11030, USA; wchaung@northwell.edu (W.C.); avarghes@northwell.edu (A.J.); zwang@northwell.edu (Z.W.); mbrenner@northwell.edu (M.B.); 2 Departments of Surgery and Molecular Medicine, Zucker School of Medicine at Hofstra/Northwell, Manhasset, NY 11030, USA

**Keywords:** total body irradiation, human ghrelin, vascular injury, endothelial cells, lungs

## Abstract

**Highlights:**

**What are the main findings?**

- Human ghrelin improves survival after total body irradiation (TBI).
- Human ghrelin’s survival benefit is in part due to the attenuation of endothelial cell permeability and barrier dysfunction in the lungs.

**What are the implications of the main findings?**

- Human ghrelin could be developed as a specific therapeutic for radiotherapy-induced vascular injury to normal tissues.
- Human ghrelin could be developed as a medical countermeasure for victims of nuclear accidents or terrorism.

**Abstract:**

Exposure of healthy tissue to ionizing radiation (IR) occurs due to nuclear accidents and terrorism, as well as radiotherapy. The vascular endothelium is a key target of IR, and microvascular endothelial cells (ECs) are particularly vulnerable to radiation. IR induces EC activation leading to endothelial cell injury. Human ghrelin is a stomach-derived peptide with pleiotropic effects, including protection against inflammation. We hypothesize that human ghrelin improves survival in total body irradiation (TBI) and that ghrelin’s protective effect could be mediated by attenuating endothelial cell injury. To test this, mice were exposed to TBI and after 24 h were treated subcutaneously with human ghrelin once daily for 4 days and monitored for 30 days. The survival rate of the human ghrelin-treated group was significantly higher than that of the vehicle group. Subsequently, human ghrelin treatment showed an effective dose modification factor of 1.0681. On day 4 after TBI, human ghrelin significantly attenuated EC permeability in the lungs and improved tight junction protein ZO-1 expression. Human ghrelin also improved ZO-1 and Claudin5 expression in primary mouse lung vascular endothelial cells. Taken together, these results indicate that human ghrelin improves survival after TBI, and its survival benefit is in part due to the attenuation of EC permeability and microvascular barrier dysfunction.

## 1. Introduction

Exposure of healthy tissue to ionizing radiation (IR) is generally inflicted by nuclear accidents and nuclear terrorism, as well as radiotherapy [1,2]. A major consequence of IR is oxidative stress resulting in acute and chronic cellular damage. The vascular endothelium is a key target of IR and microvascular endothelial cells (ECs) are particularly vulnerable to radiation [2]. Damage to these cells can be irreversible. Radiation-induced endothelial injury can occur both acutely and late after IR exposure [2]. Acute radiation syndrome (ARS) manifests in three areas, including bone marrow (H-ARS), the gastrointestinal tract (GI-ARS) and the cardiovascular/central nervous system (CNS-ARS). A few FDA-approved therapies are available for radiation injury, such as Neupogen/Neulasta or granulocyte colony-stimulating factor (G-CSF); granulocyte-macrophage colony stimulating factor (GM-CSF); and a thrombopoietin receptor agonist, Nplate (romiplostim). However, these therapies are solely effective against hematopoietic cell injury or H-ARS. Therefore, there is a growing need to develop medical countermeasures for other types of radiation-induced injury such as endothelial vascular injury.

Radiation-induced endothelial damage is associated with vascular changes leading to chronic lesions in vital organs, and patients with IR-induced tissue damage often die of organ failure [3]. Damage to endothelia is very frequent during radiation exposure in both targeted therapy and total body irradiation (TBI). The vasculature becomes leaky within hours post-IR [4]. Radiation induces changes in EC structure and function. Damage to ECs or denudation of ECs after IR produces changes in EC permeability [5,6,7] by acting on tight and adherens junctions [8]. Studies have shown that exposure of human ECs to IR significantly increased permeability and decreased transendothelial electrical resistance across the endothelial barrier [9]. Thus, IR-induced endothelial activation increases barrier dysfunction.

Human ghrelin is a 28-amino acid peptide which is predominately produced in the stomach and present at low levels in the intestine, pancreas, kidneys and placenta [10]. Ghrelin o-acyltransferase specifically acylates ghrelin at Ser3 with octanoic acid, which is essential for binding of ghrelin to the ghrelin receptor. The ghrelin receptor, a.k.a., growth hormone secretagogue receptor type 1a, is abundantly expressed in the hypothalamus and the pituitary gland, and it is also ubiquitously expressed, suggesting that ghrelin has pleiotropic activities [11,12]. Ghrelin’s broad effects include regulation of food intake, gastric motility, energy metabolism and inflammatory response [13,14,15,16]. We have previously shown that human ghrelin can effectively mitigate acute radiation injury in rats exposed to TBI [17,18]. Vascular ECs also express the ghrelin receptor and respond to ghrelin [19]. However, the effect of ghrelin on radiation-induced endothelial injury has not been elucidated. Therefore, we hypothesize that human ghrelin is protective in mice subjected to TBI and that its beneficial effect is due to inhibition of cell permeability in ECs, especially microvascular ECs. To test this, mice were exposed to TBI and treated with human ghrelin for 4 days and monitored for survival for 30 days. In additional mice exposed to TBI and human ghrelin treatment, vascular EC permeability was assessed. Finally, the effect of human ghrelin on the expression of markers of endothelial activation and cell permeability was examined in primary mouse lung vascular endothelial cells (MLVECs).

## 2. Materials and Methods

### 2.1. Experimental Animals

C57BL/6 male mice were purchased from the Jackson Laboratory (Bar Harbor, ME, USA). They were housed in a temperature-controlled room with a 12 h light/dark cycle and fed a standard Purina mouse chow diet. The mice were acclimated to the environment for 5 to 7 days. Age-matched (8–12-week-old) healthy mice were used as controls for the experiments. All experiments conducted on live animals were performed in strict adherence to the PHS policy and the Guide for the Care and Use of Laboratory Animals and were approved by the Institutional Animal Care and Use Committee of the Feinstein Institutes for Medical Research. The study was designed, conducted and reported in accordance with the ARRIVE guidelines 2.0 [20].

### 2.2. Mouse Model of Total Body Irradiation (TBI) and Treatment with Human Ghrelin

Unanesthetized male mice (10–12-week-old; 20–25 mice/group) obtained from the Jackson Laboratory or the ghrelin receptor knockout mice (Ghsr^−/−^; initially obtained from the Jackson Laboratory and bred in-house) were exposed to a single dose of 6.0 Gy TBI using an RS 2000 X-ray irradiator (Rad Source Technologies, Suwanee, GA, USA), delivered at a dose rate of approximately 1.2-Gy/min at 160 kV, 25 mA. During irradiation, the mice were gently restrained in a round plexiglass pie container (Supplementary Figure S1). After irradiation, the mice were returned to cages. The study was performed in two batches of vehicle- and human ghrelin-treated groups. The first batch consisted of 10 mice each for both groups, and the second batch included 15 mice allocated to the vehicle and 10 mice to the ghrelin treatment. A total of 10 mice were allocated to the ghrelin treatment with the assumption that ghrelin treatment would improve survival and thus achieve statistical power with a lesser number of mice. Final survival curves were determined using the combined data from the two batches of experiments (*n* = 25 for vehicle and *n* = 20 for ghrelin treatment groups). For Ghsr^−/−^, a total of 40 mice were irradiated, and they were randomly allocated to receive either vehicle (*n* = 20) or human ghrelin treatment (*n* = 20). At 24 h, the mice were randomly allocated and subcutaneously (*sc*) injected with either normal saline (vehicle) or human ghrelin (2 nmol/mouse in normal saline (Phoenix Pharmaceuticals, Burlingame, CA, USA) once daily for 4 days. The mice were monitored for 30 days, and body weight and survival time were recorded.

In addition, 12 mice were exposed to 6.0 Gy TBI, and at 24 h they were randomly allocated to either the vehicle or human ghrelin treatment (6 mice per group). An additional 5 mice were used as controls for the experiment. On day 4, lungs were harvested and flash-frozen in liquid nitrogen. Both RNA and protein were extracted for further analysis.

Post-irradiation, the mice were monitored, weighed and scored daily until the study endpoint. The mice were also monitored for humane endpoint criteria. The humane endpoint is defined as the timepoint when death is deemed imminent or suffering is irreversible. The criteria used were the following: (1) activity level is minimal or absent; (2) weight loss ≥ 20%; (3) hunched or recumbent posture; (4) minimal or no response to stimuli; (5) Grimace = 2; (6) body condition score ≤ 2. The presence of two or more of these criteria were considered as fulfilling the criteria for early euthanasia.

### 2.3. Determination of Dose Modification Factor (DMF) of Human Ghrelin After TBI

The DMF has been regarded as the best effectiveness index for radiation medical countermeasures (MCMs), and it is defined as the ratio between the dose of irradiation required for a given effect of a drug-treated group and that of the vehicle-treated group. The DMF is the essential benchmark for labeling a drug as a radiation MCM. To determine the DMF, a same-dose comparison was used. Male mice were exposed to TBI doses of 5.0, 5.5 and 6.5 Gy. Doses with 0.5 Gy increments were chosen to capture the LD_50/30_ dose for the vehicle group. At 24 h, they were treated with vehicle or human ghrelin, monitored for 30 days and survival was recorded as above. A total of 40 mice were allocated to receive 5.5 Gy, and 24 h after TBI, 20 mice each were randomly selected to receive either saline (vehicle) or human ghrelin (2 nmol/mouse in normal saline). For the 6.5 Gy group, a total of 30 mice were allocated to receive TBI, and at 24 h after TBI, 10 mice were randomly selected to receive saline (vehicle) and 20 mice were selected for human ghrelin treatment. The survival data were plotted as percent mortality vs. TBI dose, and DMF was calculated using probit analysis in Microsoft Excel 365. The 6.0 Gy data used for the DMF were sourced from the previous experiment.

### 2.4. Assessment of Vascular Permeability After TBI

To assess vascular permeability, male mice were exposed to 6.0 Gy TBI (day 0), and 24 h later they were randomly allocated for vehicle or human ghrelin treatment. AngioSense680 EX dye (2 nmol/mouse in 100 µL normal saline; Perkin Elmer, Shelton, CT, USA) was intravenously injected at day 4, and its extravasation was non-invasively quantified at day 5 by measuring the residual near-infrared fluorescence using PerkinElmer’s In Vivo Imaging System (IVIS). AngioSense is cleared from circulation within 24 h of its injection, and the residual detection of fluorescence in tissues beyond that timepoint is indicative of extravasation. There were 6 mice used as controls (non-irradiated), and 12 mice each were assigned to the TBI-Vehicle and TBI plus ghrelin groups. In a similar experiment after TBI and ghrelin treatment, at day 4, Evans Blue dye (EBD [Sigma-Aldrich, St. Louis, MO, USA]; 20 mg/kg in 100 µL normal saline) was injected intravenously, and 1 h later the mice were perfused with normal saline to remove the EBD from the blood vessels. There were 10–12 mice each assigned to the control (non-irradiated), TBI-Vehicle and TBI plus ghrelin groups. Lungs were removed, photographed, and dried at 60 °C. EBD was extracted in formamide (Sigma-Aldrich, St. Louis, MO, USA) at 37 °C for 24 h, and the amount of EBD was measured by a spectrophotometer at an OD of 620 nm.

### 2.5. Histology and Immunostaining

Male mice were exposed to 6.0 Gy TBI (day 0) and, 24 h later they were randomly allocated for treatment with either vehicle or human ghrelin. There were 3 mice each assigned to the control, TBI-Vehicle and TBI plus ghrelin groups. On day 4, the mice were euthanized, and the lungs were harvested and fixed in formalin. The fixed organs were then paraffin-embedded, sectioned into 5 µm slices, dehydrated, and stained with H&E and used for immunohistochemistry (IHC). For H&E, each tissue section was examined using a light microscope at 200× magnification. For IHC, the sections were subjected to antigen retrieval and blocked with 2% H_2_O_2_ in 60% methanol at room temperature for 10 min. Then, IgG blocking reagent in normal goat serum was applied to eliminate any nonspecific binding. The sections were stained overnight with ZO-1/TJP1 and Alexa Fluor 594 conjugate monoclonal antibody (Invitrogen/Thermo Fisher Scientific, Waltham, MA, USA), then washed and examined under a fluorescent microscope at 400× magnification.

### 2.6. Isolation of Primary Mouse Lung Vascular Endothelial Cells (MLVECs), Irradiation and Human Ghrelin Treatment

MLVECs were isolated from adult mice using a published protocol [21] with modifications. Briefly, lungs from 2–3-month-old adult mice were harvested, minced and enzymatically digested with collagenase I, and the single-cell suspension released was cultured overnight in complete endothelial cell medium (DMEM with 20% FBS, 1 × endothelial cell growth supplement, 100 μg/mL each of heparin and Pen/Strep). Then the endothelial cells were selected using anti-PECAM1 (CD-31) antibody conjugated to magnetic beads, and the cells were allowed to grow to confluence. A secondary selection was made using anti-ICAM2 (CD-102) antibody conjugated to magnetic beads, and the cells were allowed to grow to confluence. This isolation yielded approximately 1.0 × 10^6^ cells/mouse. This process takes about 7–10 days, when the MLVECs can be used for experimentation. Cultured primary MLVECs (1 × 10^5^ cells) were then exposed to 10.0 Gy radiation, treated with human ghrelin or vehicle 24 later, and harvested 48 h after treatment. A total of 10–12 mice were used for these experiments.

### 2.7. LDH Assay

LDH levels were determined using the CyQUANT LDH Cytotoxicity Assay kit (Invitrogen/Thermo Fisher Scientific, Waltham, MA, USA) according to the manufacturer’s instructions. In this step, 48 h after irradiation and human ghrelin treatment of primary MLVECs, a 50 µL aliquot of the culture medium was taken and added to the reaction mixture from the kit. After a 30 min incubation at room temperature, the reaction was interrupted by adding the stop solution from the kit, and absorbance was measured with a plate reader using ODs of 490 nm and 680 nm. LDH levels were calculated and plotted as fold changes from control samples.

### 2.8. Western Blotting

The frozen lung tissues were powdered in liquid nitrogen and homogenized in RIPA lysis buffer, and protein concentrations were estimated using the Bio Rad protein assay (BioRad, Hercules, CA, USA). Then the protein lysates (50 μg) from lung tissues and MLVECs were separated by SDS-PAGE and transferred to nitrocellulose membranes. After blocking and incubation with the respective primary antibodies, the membranes were incubated with an HRP-conjugated secondary antibody (1:10,000) (Amersham, Little Chalfont, UK) for 1 h. The membranes were then washed, and the bands reacted with ECL. They were then scanned in the Odyssey image system (LI-COR) (LI-COR Biotechnology, Lincoln, NE, USA), and densitometric analysis was quantified using ImageJ 1.× software [22].

### 2.9. Real-Time Quantitative PCR

Total RNA from lung tissues and primary MLVECs was isolated using a commercial RNA isolation kit according to manufacturer’s instructions. The purified RNA was reverse-transcribed using murine leukemia virus reverse transcriptase (Applied Biosystems, Waltham, MA, USA). PCR was carried out in a 20 µL volume containing 0.08 µM of each forward and reverse primer specific to the gene of interest, 2 µL cDNA, 7.5 µL H_2_O and 10 µL 2× SYBR Green PCR Master Mix (Applied Biosystems). Amplification was performed using the StepOne Plus Real-Time PCR System (Applied Biosystems) under the following thermal profile: 95 °C for 20 s followed by 40 cycles of 95 °C for 3 s (annealing) and 60 °C for 30 s. mRNA expression levels were quantified using the 2^−ΔΔCt^ method, with β-actin mRNA serving as the internal control. The primer sequences used for qPCR were as follows: ICAM-1: forward: 5′-GGGCTGGCATTGTTCTCTAA-3′, reverse: 5′-CTTCAGAGGCAGGAAACAGG-3′; VE-Cadherin: forward: 5′-CACTGCTTTGGGAGCCTTC-3′, reverse: 5′-GGGGCAGCGATTCATTTTTCT-3′; Claudin5: forward: 5′-GCAAGGTGTATGAATCTGTGCT-3′, reverse: 5′-GTCAAGGTAACAAAGAGTGCCA-3′; ZO-1: forward: 5′-GCCGCTAAGAGCACAGCAA-3′, reverse: 5′-TCCCCACTCTGAAAATGAGGA-3′; β-actin: forward: -5′-CGTGAAAAGATGACCCAGATCA-3′, reverse: 5′-TGGTACGACCAGAGGCATACAG-3′.

### 2.10. Statistical Analysis

Data were expressed as means ± SEs and analyzed using one-way ANOVA, followed by Student–Newman Keul’s test analysis for comparisons among multiple groups. For survival analysis, data were assessed using the Kaplan–Meier estimator and compared using the log-rank test. All statistical analyses and graphical representations were performed using GraphPad Prism Software Version 8 for Windows (www.graphpad.com).

## 3. Results

### 3.1. Human Ghrelin Improved Survival After TBI in Mice

**Figure 1:**
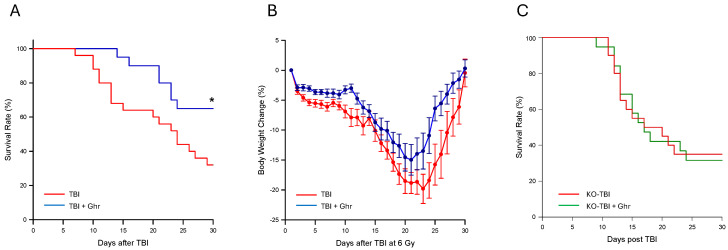
Human ghrelin improved survival after TBI. Wildtype and Ghsr^−/−^ mice were exposed to 6.0 Gy TBI and treated with vehicle or 2 nmoles of human ghrelin once daily for 4 days starting at 24 h post-TBI. (**A**) The percent changes in the 30-day survival rate and (**B**) percent changes in the bodyweight of wildtype mice are shown (*n* = 25 for vehicle; *n* = 20 for ghrelin). (**C**). The percentage changes in the survival rate of Ghsr^−/−^ 6.0 Gy TBI mice are shown (*n* = 20/group). The survival data were analyzed with the Kaplan–Meier estimator and compared using the log-rank test. * *p* < 0.05 vs. Vehicle.

The survival rate of the ghrelin-treated group was significantly higher than that of the vehicle group; the survival increased from 36% in the vehicle-treated group to 64% in the ghrelin-treated group (Figure 1A). Overall, the body weight loss of the ghrelin-treated group was less than that of the vehicle-treated group during the 30-day period after TBI (Figure 1B). In contrast, the survival curves of the ghrelin-treated group and the vehicle-treated group were similar for the Ghsr^−/−^ mice (Figure 1C). These results indicate that human ghrelin improves survival in TBI mice and that the protective effect in survival was indeed mediated by the ghrelin receptor.

### 3.2. Human Ghrelin Produced an Effective Dose Modification Factor (DMF) After TBI

**Figure 2:**
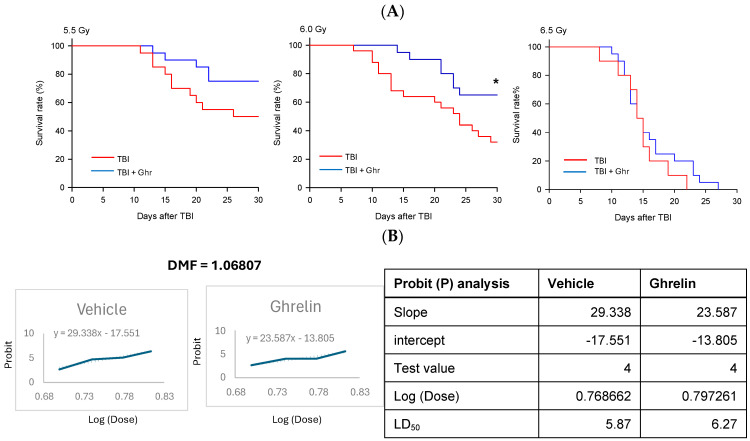
Human ghrelin produced an effective DMF after TBI. Wildtype mice were exposed to varying doses of radiation and treated with 2 nmoles of human ghrelin once daily for 4 days starting at 24 h post-TBI. (**A**) The percent changes in the 30-day survival rate of mice exposed to TBI for varying doses are shown (*n* = 20/group for 5.5 Gy TBI; *n* = 10 for vehicle and *n* = 20 for ghrelin for 6.5 Gy TBI). The data for 6.0 Gy TBI are identical to those in Figure 1A (*n* = 25 for vehicle and *n* = 20 for ghrelin). The survival data were analyzed with the Kaplan–Meier estimator and compared using the log-rank test. * *p* < 0.05 vs. Vehicle. (**B**) Probit (P) analysis was performed and DMF was calculated from the data plotted in Figure 2A.

The survival curves for the varying doses are shown (Figure 2A). Both the vehicle- and human ghrelin-treated mice had no mortality at 5 Gy. The survival curves were re-plotted as percent mortality against each irradiation dose (Figure 2B). Probit analysis using Microsoft Excel 365 was used to determine the LD_50/20_ values. Using the mortality curve, i.e., 5.0, 5.5, 6, and 6.5 Gy, the DMF was calculated as 1.0681. Thus, the treatment increased the radiation dose needed to kill 50% of the mice by at least 1 Gy, which is a critical standard requirement for GI-ARS MCMs [23].

### 3.3. Human Ghrelin Decreased Vascular Leakage After TBI

**Figure 3:**
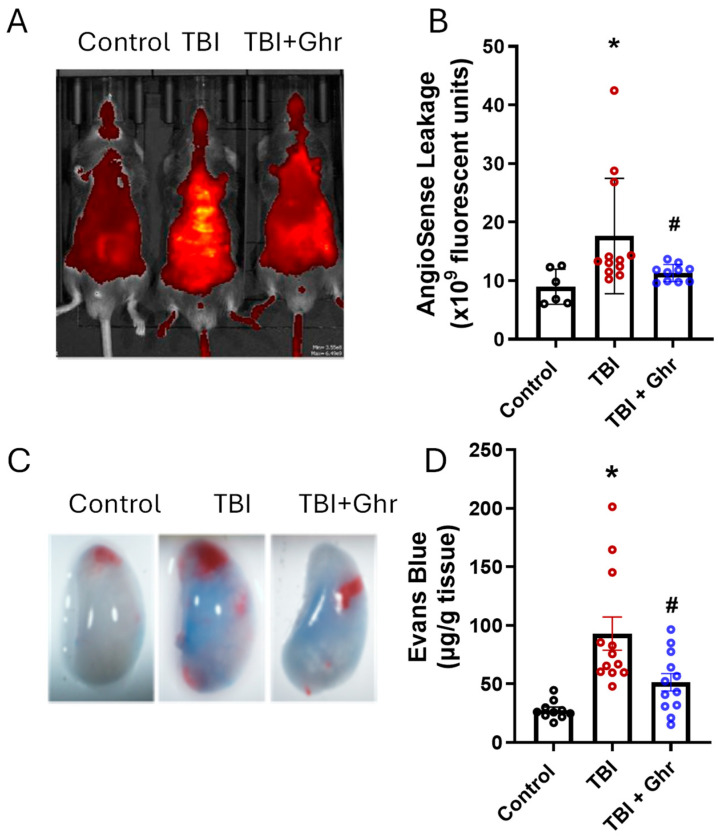
Human ghrelin decreased vascular leakage after TBI. Wildtype mice were exposed to 6.0 Gy TBI and treated with 2 nmoles of human ghrelin or normal saline once daily for 3 days starting 24 h post-TBI. The control group consisted of non-irradiated mice (**A**,**B**). AngioSense dye was intravenously injected at day 4, and its extravasation (yellow color) was quantified at day 5 using PerkinElmer’s In Vivo Imaging System (IVIS; *n* = 6 mice for control; *n* = 12 mice each for vehicle and ghrelin treatments). (**C**,**D**) At day 4 after TBI and human ghrelin treatment, Evans Blue dye (EBD) was injected intravenously, and 1 h later, mice were perfused with normal saline, lungs were harvested, EBD was extracted, and the amount of EBD was measured by a spectrophotometer and plotted (*n* = 10–12/group). The data are presented as means ± SEs and were subjected to statistical analysis through ANOVA and SNK tests. * *p* < 0.05 vs. Sham; # *p* < 0.05 vs. TBI.

In subsequent experiments, we evaluated the potential mechanisms of action of ghrelin responsible for the observed survival benefit in TBI. One such mechanism evaluated was vascular permeability, especially in the lungs. Mice exposed to TBI and treated with the vehicle showed a 2.0-fold increase in AngioSense dye extravasation compared to non-irradiated mice, whereas ghrelin treatment significantly reduced leakage by 36.7% (Figure 3A,B). Likewise, EBD retention increased by 3.7-fold in the lungs of the vehicle-treated group compared to the non-irradiated mice, while ghrelin treatment significantly decreased EBD leakage by 50.2% (Figure 3C,D).

### 3.4. Human Ghrelin Improved Vascular Integrity in the Lungs After TBI

**Figure 4:**
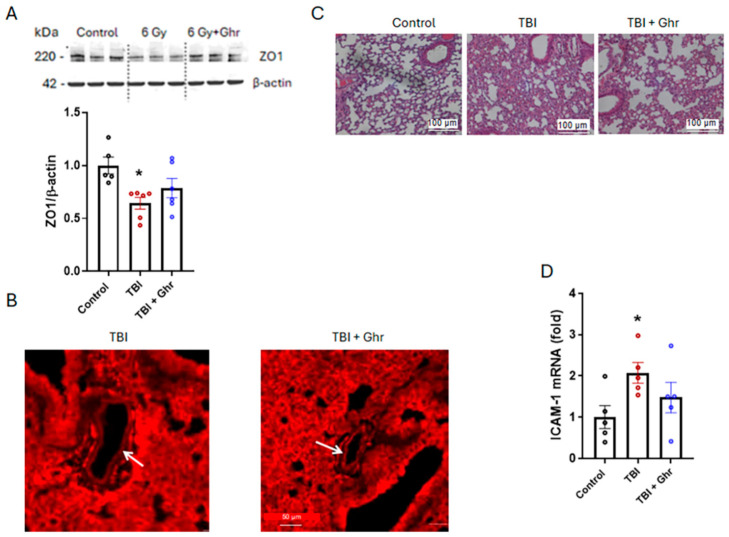
Human ghrelin improved endothelial cell integrity after TBI. Wildtype mice were exposed to 6.0 Gy TBI and treated with human ghrelin once daily for 3 days starting 24 h post-TBI. At day 4 after TBI and human ghrelin treatment, lungs were harvested. (**A**) Protein lysates were separated by SDS-PAGE and Western-blotted with ZO-1 antibody. β-actin served as internal control (*n* = 5–6 mice/group). (**B**) Lung tissue was sectioned and stained with ZO-1/TJP1 red fluorescent monoclonal antibody. The arrows are pointed to blood vessel stained in the lungs. (*n* = 3/group). The sections were examined at 400× magnification and are shown with a scale bar of 50 µm. (**C**) Lung tissue was sectioned and stained with H&E (*n* = 3 mice/group). The sections were examined at 200× magnification and are shown with a scale bar of 100 µm. (**D**) RNA was extracted and ICAM-1 mRNA was measured using quantitative real-time PCR. β-actin mRNA served as internal control (*n* = 5 mice/group). The data are presented as means ± SEs and were subjected to statistical analysis through ANOVA and SNK tests. * *p* < 0.05 vs. Sham.

Expression of tight and adherence junction proteins between endothelial cells, such as ZO-1, claudins and cadherins, are the key factors in maintenance of vascular integrity. The ZO-1 levels decreased by 37% in the vehicle-treated TBI mice, while ghrelin treatment increased the levels by 29% relative to the vehicle but did not reach statistical significance (Figure 4A). We also evaluated ZO-1 by immunofluorescence. We observed less staining in the endothelial cells after TBI, whereas ghrelin treatment showed more staining, which was nearly comparable to that of the control tissues (Figure 4B). We also evaluated the histopathology of the lung tissue and observed changes in inflammatory cell infiltration, but minimal injury was seen in the lungs (Figure 4C). These data suggest that human ghrelin treatment improved the maintenance of tight junctions of the lung vascular endothelial cells in the TBI mice. To evaluate ghrelin’s effect on endothelial activation, ICAM-1 expression was measured. ICAM-1 is a key molecule generally found on the surface of endothelial cells, especially in the pulmonary microvasculature, and it was upregulated due to IR-induced generation of reactive oxygen species [24]. ICAM-1 expression was significantly increased by 2.1-fold in the vehicle-treated mice, while ghrelin treatment reduced these levels by 29%, but the decrease did not reach statistical significance (Figure 4D). These data show that human ghrelin treatment restored the microvasculature of the lungs in the TBI mice.

### 3.5. Human Ghrelin Attenuated Endothelial Cell Permeability and Attenuated Cell Death in Irradiated MLVECs

**Figure 5:**
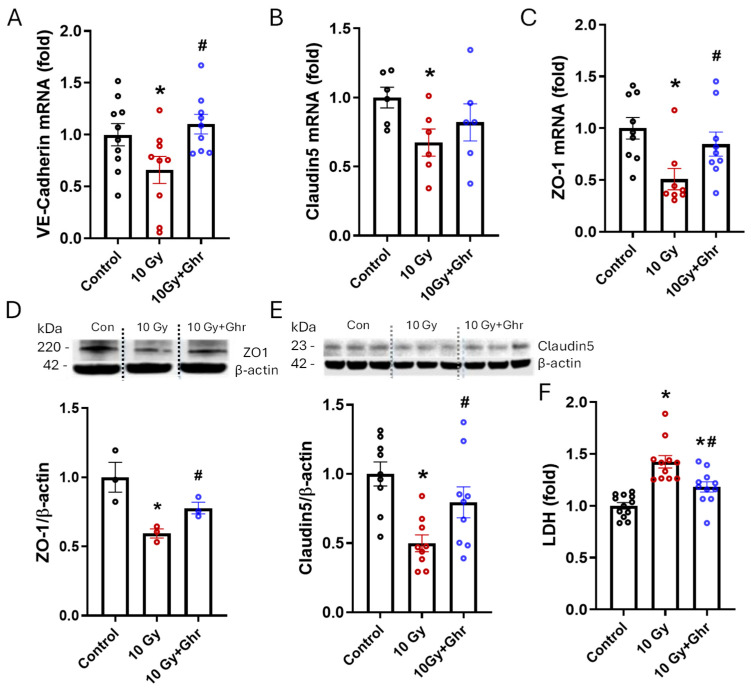
Human ghrelin decreased endothelial cell permeability and cell death in irradiated MLVECs. Primary MLVECs were exposed to 10.0 Gy radiation and treated with 1 µg/mL human ghrelin 24 h later. Cells were harvested 48 h later, and RNA was extracted. mRNA expression of VE-Cadherin (**A**), Claudin5 (**B**) and ZO-1 (**C**) mRNA was measured using quantitative real-time PCR. β-actin mRNA served as internal control. Protein lysates prepared from irradiated MLVECs treated as above were separated by SDS-PAGE and Western-blotted using ZO-1 (**D**) and Claudin5 (**E**) antibodies, and the protein expression was calculated against β-actin levels. (**F**) Irradiated cells treated with human ghrelin were assessed for extracellular LDH activity using the CyQuant LDH cytotoxicity assay kit. LDH activity is plotted as fold changes from control samples. There were 10–12 samples/group except for Claudin 5 mRNA (*n* = 6) and ZO-1 protein (*n* = 3). The data are presented as means ± SEs and were subjected to statistical analysis through ANOVA and SNK tests. * *p* < 0.05 vs. Control; # *p* < 0.05 vs. 10.0 Gy.

In primary MLVECs, VE-Cadherin mRNA decreased by 34% in the irradiated cells, whereas ghrelin treatment increased the expression by 67% and restored it to normal levels (Figure 5A). Similarly, Claudin5 mRNA decreased by 33% in the irradiated cells, while ghrelin treatment increased its expression by 22%, but there was no statistical difference between the groups (Figure 5B). ZO-1 mRNA, on the other hand, decreased in the irradiated cells by 50%, but ghrelin treatment significantly increased the expression by 66% (Figure 5C). Likewise, ZO-1 and Claudin5 proteins were decreased by 41% and 50%, respectively, in the irradiated cells, whereas ghrelin treatment significantly increased these proteins by 30% and 59% (Figure 5D,E). The effect of ghrelin on cell death induced by irradiation in MLVECs was also evaluated. The LDH levels increased by 42% in the irradiated cells, and ghrelin treatment significantly decreased the LDH levels by 83% (Figure 5F). These data indicate that human ghrelin restored endothelial cell integrity and attenuated cell death in irradiated MLVECs.

## 4. Discussion

Exposure of animals and healthy tissues to IR leads to increased production and release of oxygen radicals that result in endothelial barrier dysfunction leading to tissue injury and organ damage. Microvascular ECs are particularly sensitive to IR, and radiation-induced changes in EC function are critical components in organ damage through EC activation, increased barrier permeability and apoptosis [2]. Currently, there are no specific therapeutics or MCMs available to protect against radiation-induced loss of endothelial barrier function. Developing therapeutics against endothelial dysfunction and normal tissue damage during radiotherapy or nuclear accidents/terrorism is urgently needed as an MCM.

In the present study, we demonstrated that human ghrelin given consecutively for 4 days starting 24 h after 6.0 Gy TBI significantly improved the survival rate in mice during the 30-day observation period. To further assess the effectiveness of the human ghrelin dose used in the study, i.e., 2 nmoles/mouse, full dose–response curves from 5.0 Gy to 6.5 Gy were observed. Effectiveness was measured using the standardized method of DMF, i.e., same-dose comparison, and the DMF was calculated for LD_50/20_. Human ghrelin treatment caused a 1.0681 Gy shift in the radiation dose, thus fulfilling part of the FDA’s “animal rule” efficacy requirement for the approval of a candidate MCM [23]. In our previous rat study on TBI, we demonstrated that human ghrelin treatment attenuated gut permeability, serum endotoxin levels, bacterial translocation to the liver, intestinal apoptosis and injury and that it improved survival [18]. The current study was conducted to develop human ghrelin as an MCM for TBI. Studies on radiation MCMs are generally conducted in mice, and therefore we conducted this pre-clinical study in mice.

Since one of the major consequences of IR injury is endothelial barrier dysfunction, vascular permeability during IR and after human ghrelin treatment was evaluated. Using both Angiosense dye and EBD, we demonstrated that human ghrelin treatment significantly attenuated vascular leakage produced by IR in the whole body, especially in the lungs. In addition, IR compromises endothelial cell permeability by acting on tight and adherens junctions [8]. Tight junction adhesion is mediated by the claudin family of proteins, which are connected to the cytoskeleton by tight junction proteins, whereas adherens junctions are formed by classical cadherins that are linked to the cytoskeleton by proteins belonging to the catenin family [25,26]. In this regard, tight junction protein ZO-1 in the lungs was significantly decreased after IR, and human ghrelin treatment improved its levels by 29% but did not reach statistical significance. ICAM-1 is a key molecule upregulated due to IR-induced generation of reactive oxygen species [24]. ICAM-1 is generally found on the surface of endothelial cells, especially in the pulmonary microvasculature. The increased ICAM-1 facilitates the adherence of inflammatory cells to the endothelium and their subsequent movement into the tissues, which is essential for inflammatory response [27]. In this regard, ICAM-1 mRNA was significantly increased by IR, and human ghrelin reduced its expression by 29% but did not reach statistical significance. The lack of statistical significance observed in the treatment group for both ZO-1 and ICAM-1 could be due to the inclusion of whole-lung tissue as opposed to measurements in the endothelial cells of the lung. However, this limitation may be overcome by increasing animal numbers at the lower ghrelin dose or by increasing the ghrelin dose in future experiments, where a greater effect may be expected. To further delineate ghrelin’s direct effect on the endothelial cells in the lungs, primary MLVECs were irradiated in vitro and treated with human ghrelin. While irradiation decreased the expression of tight junction proteins such as ZO-1 and Claudin5, human ghrelin treatment increased their expression. Our in vitro studies in primary MLVECs indicate that ghrelin can in part directly act on the lung endothelium to exert its protective effects on vascular integrity. However, in vivo, it is plausible that amelioration of endotoxin release from bacteria leaking from the gut, which has a significant effect on vascular integrity, could also be the mechanism of ghrelin’s action in the lungs. In fact, our previous rat study showed that human ghrelin attenuated serum endotoxin levels and bacterial translocation to the liver, suggesting that bacterial leakage from the gut compromises vascular integrity [18]. Therefore, in the current study we extended our findings on the beneficial effects of human ghrelin not only to the survival of mice post-TBI but also to the improved vascular integrity in the lungs by attenuating radiation-induced microvascular endothelial cell permeability dysfunction.

The mechanism by which ghrelin improves vascular integrity during IR is not clearly understood. GHSR is expressed in ECs and regulates endothelial function [28,29]. Ghrelin and des-acyl ghrelin inhibit cell death in cardiomyocytes and endothelial cells through extracellular regulated protein kinases and PI3-kinase/Akt, suggesting that ghrelin, independent of its acylation, may act directly on the cardiovascular system by binding to a novel receptor distinct from GHSR-1a [28]. Ghrelin inhibits basal and TNF-α-induced cytokine production and mononuclear cell adhesion in human vascular endothelial cells in vitro [29]. In vivo, intravenous treatment with ghrelin inhibits cytokine release induced by endotoxemia. This study suggests that these anti-inflammatory effects help explain the protective effect of ghrelin in myocardial reperfusion injury, cardiac cachexia and septic shock. In fact, ghrelin inhibits NF-kB activation in endothelial cells, suggesting a potential mechanism of action for its anti-inflammatory effects [29]. In another study, ghrelin stimulated endothelial cell angiogenesis through the extracellular-regulated protein kinase signaling pathway, and this effect was mediated by the GHSR-1a receptor [30]. Endothelial GHSR is important in regulating lipid metabolism in response to ghrelin, and targeting endothelial ghrelin signaling could manage excessive adiposity and associated metabolic disorders [31]. However, additional studies are needed to elucidate the actual mechanism of action of human ghrelin on vascular integrity after TBI.

How ghrelin attenuates radiation-induced lung inflammation has also not been completely elucidated. Signaling pathways involving ghrelin/GHSR-1a are complex. As a G protein-coupled receptor, GHSR-1a initiates multiple intracellular signaling pathways, including the activation of phospholipase Cγ, β-arrestin and inositol phosphatase 3 kinase cascades [32,33]. For instance, upon GHSR-1a stimulation, phosphorylated extracellular-regulated protein kinase activation via the β-arrestin pathway and protein kinase B (Akt) phosphorylation via elevated PI3K activity are observed [34]. Ghrelin regulates inflammation in neurological, cardiovascular, hepatic, gastrointestinal, kidney and respiratory diseases [35]. Regarding the lungs, ghrelin pretreatment ameliorates mechanical ventilation-induced pulmonary inflammation by decreasing cytokine release and suppressing the TLR4/NF-kB pathway [36]. Another study suggests that GHSR-1a deletion inhibits lung inflammation and the disruption of the alveolar barrier in the LPS-induced acute respiratory distress syndrome model [37]. Likewise, ghrelin administration in sepsis-induced acute lung injury in a rat model of sepsis inhibits the NF-kB/iNOS and Akt signaling pathways [38]. Ghrelin exerts an anti-apoptotic effect on alveolar macrophages by inhibiting C-Jun Kinase and activating the Wnt/β-catenin pathway, which helps to alleviate sepsis-induced ARDS [39]. Ghrelin has been regarded as a vasodilatory peptide. Intravenous administration of ghrelin in healthy volunteers produced a significant reduction in peripheral vascular resistance and increased cardiac output without any significant changes in heart rate. These cardiovascular responses mimic the hyperdynamic phase that is generally observed in polymicrobial sepsis [40].

Prior studies by us showed that ghrelin levels in circulation decreased 5 h (hyperdynamic phase) and 20 h (hypodynamic phase) after sepsis [41]. Similar decreases in plasma levels of ghrelin were also shown in an endotoxemia model [42]. Intravenous administration of ghrelin starting 5 h after sepsis markedly reduced plasma cytokine levels, providing strong evidence that ghrelin could be a beneficial therapeutic agent in reducing inflammation. We have further shown that ghrelin’s inhibition of inflammation in sepsis is mediated by the combined action of the inhibition of the sympathetic nervous system and the activation of the vagus nerve [15,16]. Furthermore, we have shown that plasma ghrelin levels were markedly decreased in a radiation combined injury (RCI) model of TBI followed by sepsis. Intravenous administration of ghrelin in RCI produced a significant decrease in plasma cytokine levels and gut tissue levels of cytokines and a decrease in myeloperoxidase activities of the gut, lungs and kidneys, demonstrating that ghrelin’s protective effect in RCI is mediated by inhibition of the sympathetic nervous system [17]. In addition, we have shown that prior vagotomy in RCI rats abolished ghrelin’s protective effect in the inflammatory response, suggesting that ghrelin’s effect in RCI is attributable to balancing of the dysregulated sympathetic and parasympathetic nervous systems caused by injuries [17]. Thus, a myriad of mechanisms has been reported for ghrelin’s protective effect in lung inflammation. However, future studies are warranted to elucidate the actual mechanism of action of ghrelin in radiation-induced lung inflammation.

The concentration of acylated ghrelin in circulation is around 9 ng/mL in rats. Although we did not measure plasma ghrelin in the current study, we have previously shown that 7 days post-TBI, plasma ghrelin was significantly reduced by 27% from the control value [18]. Therefore, it is highly likely that ghrelin levels could be decreased earlier than 7 days post-TBI. Further studies are needed for confirmation.

While our study provides valuable insights into the potential of human ghrelin as a countermeasure for radiation-induced endothelial injury, we acknowledge several limitations that warrant consideration and should guide future research. First, our mechanistic investigations primarily focused on lung endothelial cells (ECs). We specifically selected lung ECs due to their ease of isolation for detailed in vitro and ex vivo analyses compared with other radiosensitive organs, such as the intestine, allowing us to effectively model endothelial cell permeability and barrier dysfunction following total body irradiation (TBI). We recognize that at early timepoints (e.g., 4 days post-6.0 Gy TBI), overt injury in specific organs like the lungs or the gut may not be histopathologically apparent. However, endothelial dysfunction is an early and systemic event following TBI. Importantly, our in vivo imaging system (IVIS) using Angiosense 680EX allowed for the assessment of extravasation and vascular permeability across the entire body, including other radiosensitive organs such as the gut, thereby providing a more global measure of ghrelin’s impact on vascular integrity. Second, while our primary focus was on the effects of human ghrelin on IR-induced endothelial cell permeability, the complex cascade of endothelial vascular injury involves multiple additional pathways. These include, but are not limited to, generalized inflammatory responses, mitochondrial dysfunction, and various forms of programmed cell death [43,44,45]. It has been shown that ICAM-1 was upregulated by irradiation in vivo and that administration of an anti-ICAM-1 antibody modulated the resulting leukocyte adhesion [46]. Indeed, we demonstrated that human ghrelin attenuated the upregulation of ICAM-1 by 29%. This finding, consistent with our previous work showing ghrelin’s anti-inflammatory effects in experimental sepsis [15], suggests ghrelin’s potential to broadly modulate IR-induced EC inflammatory responses. Future studies are warranted to comprehensively investigate ghrelin’s impact on other critical aspects of IR-induced endothelial damage, such as mitochondrial function and specific cell death pathways like pyroptosis triggered by activation of inflammasome signaling [47,48,49] which our lab has previously identified as a key mechanism in EC injury [50] and has been implicated in radiation-induced endothelial damage [51]. Third, based on prior experience with ghrelin in inflammatory conditions [18], we utilized a single dose (2 nmoles/mouse) of human ghrelin in the current study. Comprehensive dose–response and time-course studies are crucial for optimizing ghrelin’s therapeutic application against IR-induced endothelial vascular injury. Finally, our study only used male mice. Given established sex differences in physiological responses and disease susceptibility, future investigations will need to include female mice to fully assess the generalizability and optimize ghrelin’s therapeutic potential, particularly when considering dose and time responses.

## 5. Conclusions

In conclusion, human ghrelin significantly improved survival and EC barrier dysfunction following radiation exposure. Thus, human ghrelin could be developed in the future as a therapeutic for radiotherapy-induced damage to normal tissues, as well as mass casualty events stemming from nuclear accidents or radiological terrorism.

## Data Availability

The original contributions presented in this study are included in the article/Supplementary Materials. Further inquiries can be directed to the corresponding author.

## Supplementary Materials

The following supporting information can be downloaded at: https://www.mdpi.com/article/10.3390/cells15070586/s1, Supplementary Figure S1: Photograph of the device used to gently restrain the mice during radiation.

## Abbreviations

The following abbreviations are used in this manuscript:

IR	Ionizing radiation
ECs	Endothelial cells
TBI	Total body irradiation
ARS	Acute radiation syndrome
DMF	Dose modification factor
EBD	Evan’s blue dye
MLVECs	Mouse lung vascular endothelial cells
LDH	Lactate dehydrogenase
MCM	Medical countermeasure

## References

[1] Singh V.K., Seed T.M. (2017). A review of radiation countermeasures focusing on injury-specific medicinals and regulatory approval status: Part I. Radiation sub-syndromes, animal models and FDA-approved countermeasures. Int. J. Radiat. Biol..

[2] Wijerathne H., Langston J.C., Yang Q., Sun S., Miyamoto C., Kilpatrick L.E., Kiani M.F. (2021). Mechanisms of radiation-induced endothelium damage: Emerging models and technologies. Radiother. Oncol..

[3] Satyamitra M.M., DiCarlo A.L., Taliaferro L. (2016). Understanding the Pathophysiology and Challenges of Development of Medical Countermeasures for Radiation-Induced Vascular/Endothelial Cell Injuries: Report of a NIAID Workshop, August 20, 2015. Radiat. Res..

[4] Sharma P., Templin T., Grabham P. (2013). Short term effects of gamma radiation on endothelial barrier function: Uncoupling of PECAM-1. Microvasc. Res..

[5] Maj J.G., Paris F., Haimovitz-Friedman A., Venkatraman E., Kolesnick R., Fuks Z. (2003). Microvascular function regulates intestinal crypt response to radiation. Cancer Res..

[6] Paris F., Fuks Z., Kang A., Capodieci P., Juan G., Ehleiter D., Haimovitz-Friedman A., Cordon-Cardo C., Kolesnick R. (2001). Endothelial apoptosis as the primary lesion initiating intestinal radiation damage in mice. Science.

[7] Rotolo J.A., Maj J.G., Feldman R., Ren D., Haimovitz-Friedman A., Cordon-Cardo C., Cheng E.H., Kolesnick R., Fuks Z. (2008). Bax and Bak do not exhibit functional redundancy in mediating radiation-induced endothelial apoptosis in the intestinal mucosa. Int. J. Radiat. Oncol. Biol. Phys..

[8] Kabacik S., Raj K. (2017). Ionising radiation increases permeability of endothelium through ADAM10-mediated cleavage of VE-cadherin. Oncotarget.

[9] Soroush F., Tang Y., Zaidi H.M., Sheffield J.B., Kilpatrick L.E., Kiani M.F. (2018). PKCdelta inhibition as a novel medical countermeasure for radiation-induced vascular damage. FASEB J..

[10] Kojima M., Hosoda H., Date Y., Nakazato M., Matsuo H., Kangawa K. (1999). Ghrelin is a growth-hormone-releasing acylated peptide from stomach. Nature.

[11] Papotti M., Ghe C., Cassoni P., Catapano F., Deghenghi R., Ghigo E., Muccioli G. (2000). Growth hormone secretagogue binding sites in peripheral human tissues. J. Clin. Endocrinol. Metab..

[12] Gnanapavan S., Kola B., Bustin S.A., Morris D.G., McGee P., Fairclough P., Bhattacharya S., Carpenter R., Grossman A.B., Korbonits M. (2002). The tissue distribution of the mRNA of ghrelin and subtypes of its receptor, GHS-R, in humans. J. Clin. Endocrinol. Metab..

[13] Druce M.R., Wren A.M., Park A.J., Milton J.E., Patterson M., Frost G., Ghatei M.A., Small C., Bloom S.R. (2005). Ghrelin increases food intake in obese as well as lean subjects. Int. J. Obes..

[14] Nakazato M., Murakami N., Date Y., Kojima M., Matsuo H., Kangawa K., Matsukura S. (2001). A role for ghrelin in the central regulation of feeding. Nature.

[15] Wu R., Dong W., Cui X., Zhou M., Simms H.H., Ravikumar T.S., Wang P. (2007). Ghrelin down-regulates proinflammatory cytokines in sepsis through activation of the vagus nerve. Ann. Surg..

[16] Wu R., Zhou M., Das P., Dong W., Ji Y., Yang D., Miksa M., Zhang F., Ravikumar T.S., Wang P. (2007). Ghrelin inhibits sympathetic nervous activity in sepsis. Am. J. Physiol. Endocrinol. Metab..

[17] Shah K.G., Wu R., Jacob A., Blau S.A., Ji Y., Dong W., Marini C.P., Ravikumar T.S., Coppa G.F., Wang P. (2009). Human ghrelin ameliorates organ injury and improves survival after radiation injury combined with severe sepsis. Mol. Med..

[18] Wang Z., Yang W.L., Jacob A., Aziz M., Wang P. (2015). Human ghrelin mitigates intestinal injury and mortality after whole body irradiation in rats. PLoS ONE.

[19] Tesauro M., Schinzari F., Caramanti M., Lauro R., Cardillo C. (2010). Cardiovascular and metabolic effects of ghrelin. Curr. Diabetes Rev..

[20] Percie du Sert N., Hurst V., Ahluwalia A., Alam S., Avey M.T., Baker M., Browne W.J., Clark A., Cuthill I.C., Dirnagl U. (2020). The ARRIVE guidelines 2.0: Updated guidelines for reporting animal research. PLoS Biol..

[21] Wong E., Nguyen N., Hellman J. (2021). Isolation of Primary Mouse Lung Endothelial Cells. J. Vis. Exp..

[22] Schneider C.A., Rasband W.S., Eliceiri K.W. (2012). NIH Image to ImageJ: 25 years of image analysis. Nat. Methods.

[23] Williams J.P., Brown S.L., Georges G.E., Hauer-Jensen M., Hill R.P., Huser A.K., Kirsch D.G., Macvittie T.J., Mason K.A., Medhora M.M. (2010). Animal models for medical countermeasures to radiation exposure. Radiat. Res..

[24] Hallahan D.E., Virudachalam S. (1997). Ionizing radiation mediates expression of cell adhesion molecules in distinct histological patterns within the lung. Cancer Res..

[25] Dejana E., Vestweber D. (2013). The role of VE-cadherin in vascular morphogenesis and permeability control. Prog. Mol. Biol. Transl. Sci..

[26] Steed E., Balda M.S., Matter K. (2010). Dynamics and functions of tight junctions. Trends Cell Biol..

[27] Bui T.M., Wiesolek H.L., Sumagin R. (2020). ICAM-1: A master regulator of cellular responses in inflammation, injury resolution, and tumorigenesis. J. Leukoc. Biol..

[28] Baldanzi G., Filigheddu N., Cutrupi S., Catapano F., Bonissoni S., Fubini A., Malan D., Baj G., Granata R., Broglio F. (2002). Ghrelin and des-acyl ghrelin inhibit cell death in cardiomyocytes and endothelial cells through ERK1/2 and PI 3-kinase/AKT. J. Cell Biol..

[29] Li W.G., Gavrila D., Liu X., Wang L., Gunnlaugsson S., Stoll L.L., McCormick M.L., Sigmund C.D., Tang C., Weintraub N.L. (2004). Ghrelin inhibits proinflammatory responses and nuclear factor-kappaB activation in human endothelial cells. Circulation.

[30] Wang L., Chen Q., Pang J. (2023). The effects and mechanisms of ghrelin upon angiogenesis in human coronary artery endothelial cells under hypoxia. Peptides.

[31] Urai H., Azegami T., Komatsu M., Takahashi R., Kubota Y., Hasegawa K., Tokuyama H., Wakino S., Hayashi K., Kanda T. (2024). Ghrelin Promotes Lipid Uptake into White Adipose Tissue via Endothelial Growth Hormone Secretagogue-Receptor in Mice. Nutrients.

[32] Huang J., Liu W., Doycheva D.M., Gamdzyk M., Lu W., Tang J., Zhang J.H. (2019). Ghrelin attenuates oxidative stress and neuronal apoptosis via GHSR-1alpha/AMPK/Sirt1/PGC-1alpha/UCP2 pathway in a rat model of neonatal HIE. Free Radic. Biol. Med..

[33] Smith R.G. (2016). Dissociating ghrelin-dependent G protein from beta-arrestin-2 signaling in transgenic rats. Sci. Signal..

[34] Yuan M.J., Wang T. (2020). The new mechanism of Ghrelin/GHSR-1a on autophagy regulation. Peptides.

[35] Ma Y., Zhang H., Guo W., Yu L. (2022). Potential role of ghrelin in the regulation of inflammation. FASEB J..

[36] Li G., Liu J., Xia W.F., Zhou C.L., Lv L.Q. (2017). Protective effects of ghrelin in ventilator-induced lung injury in rats. Int. Immunopharmacol..

[37] Tanida R., Tsubouchi H., Yanagi S., Saito Y., Toshinai K., Miyazaki T., Takamura T., Nakazato M. (2022). GHS-R1a deficiency mitigates lipopolysaccharide-induced lung injury in mice via the downregulation of macrophage activity. Biochem. Biophys. Res. Commun..

[38] Zheng H., Liang W., He W., Huang C., Chen Q., Yi H., Long L., Deng Y., Zeng M. (2019). Ghrelin attenuates sepsis-induced acute lung injury by inhibiting the NF-kappaB, iNOS, and Akt signaling in alveolar macrophages. Am. J. Physiol. Lung Cell. Mol. Physiol..

[39] Li B., Zeng M., He W., Huang X., Luo L., Zhang H., Deng D.Y. (2015). Ghrelin protects alveolar macrophages against lipopolysaccharide-induced apoptosis through growth hormone secretagogue receptor 1a-dependent c-Jun N-terminal kinase and Wnt/beta-catenin signaling and suppresses lung inflammation. Endocrinology.

[40] Okumura H., Nagaya N., Enomoto M., Nakagawa E., Oya H., Kangawa K. (2002). Vasodilatory effect of ghrelin, an endogenous peptide from the stomach. J. Cardiovasc. Pharmacol..

[41] Wu R., Zhou M., Cui X., Simms H.H., Wang P. (2004). Upregulation of cardiovascular ghrelin receptor occurs in the hyperdynamic phase of sepsis. Am. J. Physiol. Heart Circ. Physiol..

[42] Basa N.R., Wang L., Arteaga J.R., Heber D., Livingston E.H., Tache Y. (2003). Bacterial lipopolysaccharide shifts fasted plasma ghrelin to postprandial levels in rats. Neurosci. Lett..

[43] Guipaud O., Jaillet C., Clement-Colmou K., Francois A., Supiot S., Milliat F. (2018). The importance of the vascular endothelial barrier in the immune-inflammatory response induced by radiotherapy. Br. J. Radiol..

[44] Venkatesulu B.P., Mahadevan L.S., Aliru M.L., Yang X., Bodd M.H., Singh P.K., Yusuf S.W., Abe J.I., Krishnan S. (2018). Radiation-Induced Endothelial Vascular Injury: A Review of Possible Mechanisms. JACC Basic Transl. Sci..

[45] Wirsdorfer F., Jendrossek V. (2016). The Role of Lymphocytes in Radiotherapy-Induced Adverse Late Effects in the Lung. Front. Immunol..

[46] Yuan H., Gaber M.W., McColgan T., Naimark M.D., Kiani M.F., Merchant T.E. (2003). Radiation-induced permeability and leukocyte adhesion in the rat blood-brain barrier: Modulation with anti-ICAM-1 antibodies. Brain Res..

[47] Bergsbaken T., Fink S.L., Cookson B.T. (2009). Pyroptosis: Host cell death and inflammation. Nat. Rev. Microbiol..

[48] Martinon F., Burns K., Tschopp J. (2002). The inflammasome: A molecular platform triggering activation of inflammatory caspases and processing of proIL-beta. Mol. Cell.

[49] Xiang M., Shi X., Li Y., Xu J., Yin L., Xiao G., Scott M.J., Billiar T.R., Wilson M.A., Fan J. (2011). Hemorrhagic shock activation of NLRP3 inflammasome in lung endothelial cells. J. Immunol..

[50] Yang W.L., Sharma A., Wang Z., Li Z., Fan J., Wang P. (2016). Cold-inducible RNA-binding protein causes endothelial dysfunction via activation of Nlrp3 inflammasome. Sci. Rep..

[51] Smith A.O., Ju W., Adzraku S.Y., Wenyi L., Yuting C., Qiao J., Xu K., Zeng L. (2021). Gamma Radiation Induce Inflammasome Signaling and Pyroptosis in Microvascular Endothelial Cells. J. Inflamm. Res..

