# Supplementary figures and images for: Human Ghrelin Improves Vascular Integrity and Survival After Total Body Irradiation
Source: Cells. 2026 Mar 26;15(7):586. doi: 10.3390/cells15070586 (PMC13072092; doi:10.3390/cells15070586)

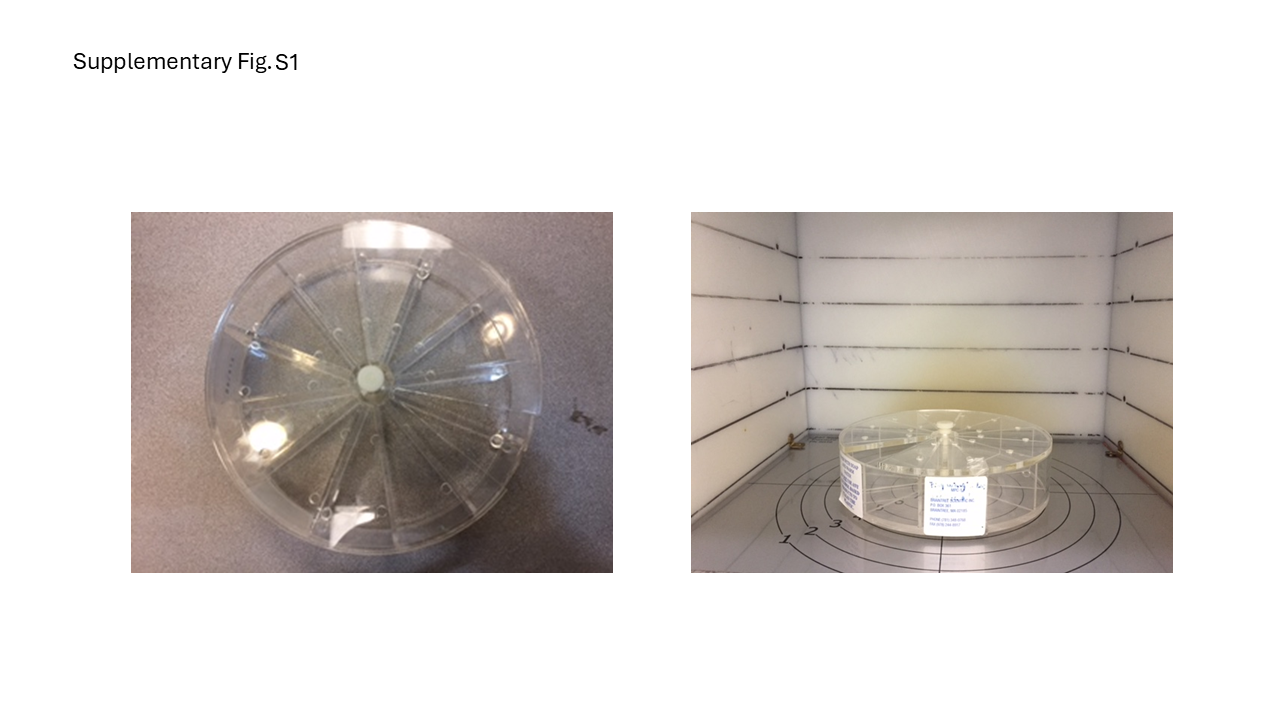

Supplement: Supplementary file 1 [file cells-15-00586-s001.zip › cells-4198216-supplementary.tif]
